# Mouse Gestation Length Is Genetically Determined

**DOI:** 10.1371/journal.pone.0012418

**Published:** 2010-08-25

**Authors:** Stephen A. Murray, Judith L. Morgan, Coleen Kane, Yashoda Sharma, Caleb S. Heffner, Jeffrey Lake, Leah Rae Donahue

**Affiliations:** The Jackson Laboratory, Bar Harbor, Maine, United States of America; Washington University, United States of America

## Abstract

**Background:**

Preterm birth is an enormous public health problem, affecting over 12% of live births and costing over $26 billion in the United States alone. The causes are complex, but twin studies support the role of genetics in determining gestation length. Despite widespread use of the mouse in studies of the genetics of preterm birth, there have been few studies that actually address the precise natural gestation length of the mouse, and to what degree the timing of labor and birth is genetically determined.

**Methodology/Principal Findings:**

To further develop the mouse as a genetic model of preterm birth, we developed a high-throughput monitoring system and measured the gestation length in 15 inbred strains. Our results show an unexpectedly wide variation in overall gestation length between strains that approaches two full days, while intra-strain variation is quite low. Although litter size shows a strong inverse correlation with gestation length, genetic difference alone accounts for a significant portion of the variation. In addition, ovarian transplant experiments support a primary role of maternal genetics in the determination of gestation length. Preliminary analysis of gestation length in the C57BL/6J-Chr#^A/J^/NaJ chromosome substitution strain (B.A CSS) panel suggests complex genetic control of gestation length.

**Conclusions/Significance:**

Together, these data support the role of genetics in regulating gestation length and present the mouse as an important tool for the discovery of genes governing preterm birth.

## Introduction

More than 12% of infants are born prematurely and suffer a high degree of morbidity and mortality (www.marchofdimes.com/peristats/). The cost to the healthcare system to care for these infants is enormous, estimated to be at least $26.2 billion per year in the U.S. alone [1]. Twin and association studies in humans have demonstrated that there is a strong genetic contribution to the determination of gestation time, but specific causative genes have not been identified [2]. The mouse is an excellent choice to model many complex human diseases and has been successfully exploited for gene discovery. Although the mouse has been used to model some aspects of preterm birth [3], [4], many basic features of mouse physiology as it relates to parturition remain unclear. One critical gap in our understanding is the precise gestation length (GL) of the mouse, generally thought to be between 18 and 22 days. It is not clear if this range represents intra- or inter-strain variability, if reported differences in gestation time are dependent of litter size, or if length of gestation affects pup survival. If the mouse is to serve as a useful tool for the study of preterm birth, we need precise information about the normal course of pregnancy in mice. Moreover, identifying genes that control differences in GL in rodents can facilitate gene identification in large scale human genetic association studies currently underway [5].

## Results

**Figure 1:**
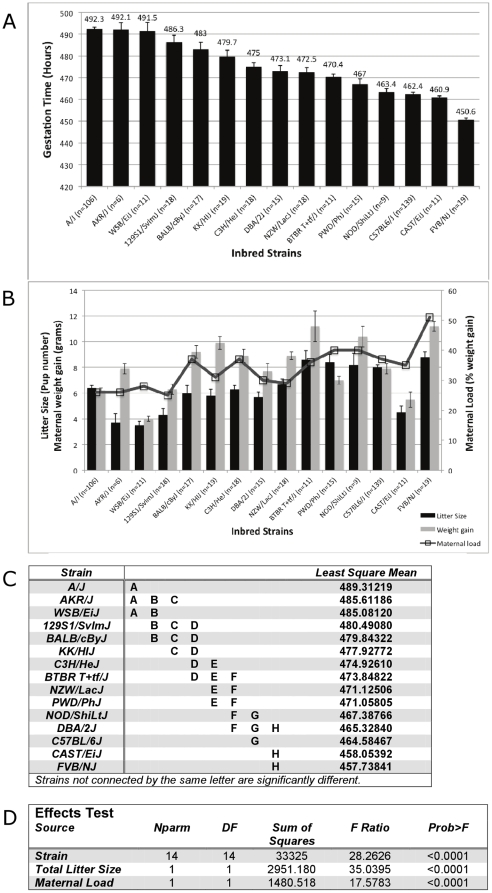
Highly significant differences in gestation length among inbred mouse strains. (A) Gestational length presented in total hours, measured from the midpoint of the dark cycle prior to the appearance of a copulation plug to the recorded appearance of the first pup. The total number of pregnancies monitored is indicated for each strain and data are presented as the mean +/− S.E.M. A detailed description of the animal husbandry and measurement procedures is provided in the supplementary methods (6). (B) Live litter size (number of pups), maternal weight gain (at E14.5) and maternal load (% weight gain) for each of the 15 inbred strains measured in (A). Complete data are presented in Table 1. (C) Effects test (analysis of covariance) demonstrating significant differences in GL among inbred strains independent of the effect of total litter size and maternal load. Litter size is also significantly different among strains and is strongly correlated with GL. (D) Graphical representation of strains with significantly different GLs independent of all other factors. Strains that are not associated by a letter are significantly different from each other (p<0.05).

To develop the mouse as a tool for investigating the genetic regulation of GL, we have developed a high-throughput phenotyping platform to precisely measure the time of birth in mice. This system is comprised of an array of closed circuit, infrared-sensitive cameras arranged to constantly monitor individual pregnant females, and allows us to ascertain the delivery of the first pup within a window of five minutes. A natural, timed mating scheme was used to initiate pregnancy, allowing us to estimate the start of gestation to be the mid-point of the dark cycle prior to the appearance of the copulation plug. Using this system, we determined the gestation time of 15 different inbred strains of mice. These strains display wide genetic diversity, are associated with extensive phenotypic data (Mouse Phenome Database (www.phenome.jax.org), and have been re-sequenced to provide high-density SNP maps [6]. As shown in Figure 1A, the GL for individual strains varies from 451 hours to 493 hours, or nearly two full days. Moreover, the GL within an individual strain is remarkably consistent, strongly supporting a direct role for genetics in determining GL in mice. Although we did not attempt to measure the precise time of mating and conception, the relatively low level of variability in our results suggest consistent mating behavior within a given strain. Pup survival in the early postnatal period is variable among strains, ranging from 100% for several strains to as low as 60% for WSB/EiJ (Table 1). However, this does not appear to be related to GL, as strains with both short (CAST/EiJ) and long (WSB/EiJ and A/J) GLs display some degree of pup mortality (Table 1). Despite living in a highly controlled environment, we observed that C57BL/6J (B6) exhibit significant seasonal variation in their overall GL, specifically a shortening of the GL during the fall (456.6 hours in the fall versus 462.4 hours, 467.6 hours and 465.2 hours in the winter, spring and summer, respectively). This seasonal effect is consistent with findings for other mouse phenotypes, including bone mineral density [7]. However, the low variability in the year-long B6 dataset suggests the impact of this variation on the total analysis is minimal. Given the wide variation between inbred strains and small amount of intra-strain variability, these data clearly show that genetics has a major role in the length of natural gestation time.

**Table 1:** Data summary from individual strain measurements.

Strain	Gestation Time (Hours)	Litter Size	Weight per live pup (grams)	Survival Rate (%)	Maternal Weight (grams)	Weight Gain at E14.5 (grams)	Pregnancy Load (% weight gain)
***129S1/SvImJ***	486.3+/−3.2*n = 18*	4.3+/−0.5*n = 18*	1.6+/−0.04*n = 15*	87%	25.1+/−0.4*n = 18*	6.3+/−0.4*n = 18*	25%
***A/J***	492.3+/−0.9*n = 106*	6.4+/−0.2*n = 106*	1.3+/−0.01*n = 90*	67%	23.9+/−0.2*n = 100*	6.2+/−0.2*n = 100*	26%
***AKR/J***	492.1+/−3.2n = 6	3.7+/−0.7n = 6	1.5+/−0.03n = 6	95%	30.5+/−0.7n = 6	7.9+/−0.4n = 6	26%
***C57BL/6J***	462.4+/−1.0n = 139	8.0+/−0.2n = 138	1.4+/−0.01n = 138	97%	25.3+/−0.2n = 124	7.9+/−0.4n = 124	37%
***BALB/cByJ***	483+/−3.3n = 17	6.0+/−0.6n = 17	1.6+/−0.04n = 16	98%	24.8+/−0.4n = 14	9.2+/−0.5n = 14	37%
***BTBR T+tf/J***	470.4+/−1.2n = 11	8.6+/−0.7n = 11	1.6+/−0.07n = 11	99%	24.4+/−1.1n = 11	11.2+/−1.2n = 11	36%
***C3H/HeJ***	475+/−1.9n = 18	6.3+/−0.3n = 18	1.4+/−0.03n = 18	95%	24.4+/−0.5n = 17	8.9+/−0.5n = 17	37%
***CAST/EiJ***	460.9+/−0.7n = 11	4.5+/−0.5n = 11	1.1+/−0.02n = 10	78%	15.5+/−0.3n = 11	5.5+/−0.6n = 11	35%
***DBA/2J***	473.1+/−2.5n = 15	5.7+/−0.4n = 15	1.3+/−0.02n = 13	97%	25.5+/−1.1n = 6	7.7+/−0.6n = 6	30%
***FVB/NJ***	450.6+/−0.8n = 19	8.8+/−0.4n = 19	1.4+/−0.02n = 19	100%	22.5+/−0.3n = 16	11.2+/−0.4n = 16	51%
***KK/HIJ***	479.7+/−2.9n = 19	5.8+/−0.5n = 19	1.3+/−0.02n = 19	72%	31.8+/−1.1n = 19	9.9+/−0.5n = 19	31%
***NOD/ShiLtJ***	463.4+/−1.6n = 9	8.2+/−0.9n = 9	1.4+/−0.07n = 9	86%	26.0+/−0.5n = 9	10.4+/−0.8n = 9	40%
***NZW/LacJ***	472.5+/−2.2n = 18	6.7+/−0.4n = 18	1.5+/−0.03n = 18	99%	30.6+/−0.5n = 17	8.9+/−0.3n = 17	29%
***PWD/PhJ***	467+/−2.4n = 15	8.4+/−0.6n = 15	1.1+/−0.01n = 15	100%	17.8+/−0.3n = 15	7.0+/−0.3n = 15	40%
***WSB/EiJ***	491.5+/−3.9n = 11	3.5+/−0.3n = 11	1.2+/−0.08n = 7	60%	14.7+/−0.5n = 11	4.0+/−0.2n = 11	28%

Total litter size includes all (live and dead) pups identified. Weight per live pup is the average of all surviving pups for each individual strain. Dead pups were often found desiccated and partially cannibalized and are thus excluded. Survival rate is the percentage of total pups identified that survived until at least postnatal day 3. Maternal weight was measured and recorded following the identification of a copulation plug and at E14.5 to calculate weight gain. At this time point, females were housed in front of the cameras and not disturbed until a birth was recorded. Pregnancy load is the maternal weight gained as a percentage of initial weight following a successful mating.

In the human population, women carrying multiple fetuses are at significantly higher risk for preterm delivery [1]. To determine if a similar correlation is seen in mice, we documented both the total number of pups (Figure 1B and Table 1) and the total weight of live pups (Table 1) born in each litter. Because the maternal size, and therefore potentially the ability to carry large number of pups, varies significantly between inbred strains (Table 1), we also recorded the weight gain of the mother through E14.5, at which point they were placed in front of the cameras and left undisturbed. Weight gain was then normalized as a percentage of pre-pregnancy body weight (“pregnancy load”; see Methods for details) (Figure 1B and Table 1). We observe a strong inverse correlation between GL and litter size (R = −0.62, p<0.001,), between GL and maternal weight gain (R = −0.56, p<0.001) and between GL and overall pregnancy load (R = −0.58, p<0.001). Using an effects test, however, we find that gestation time itself, irrespective of the litter size effect, is significantly different among many of the 15 strains tested (Figure 1C, D). Therefore, although litter size and relative fetal burden is clearly inversely correlated with GL, strain genetics has a highly significant and independent role in determining total GL.

**Figure 2:**
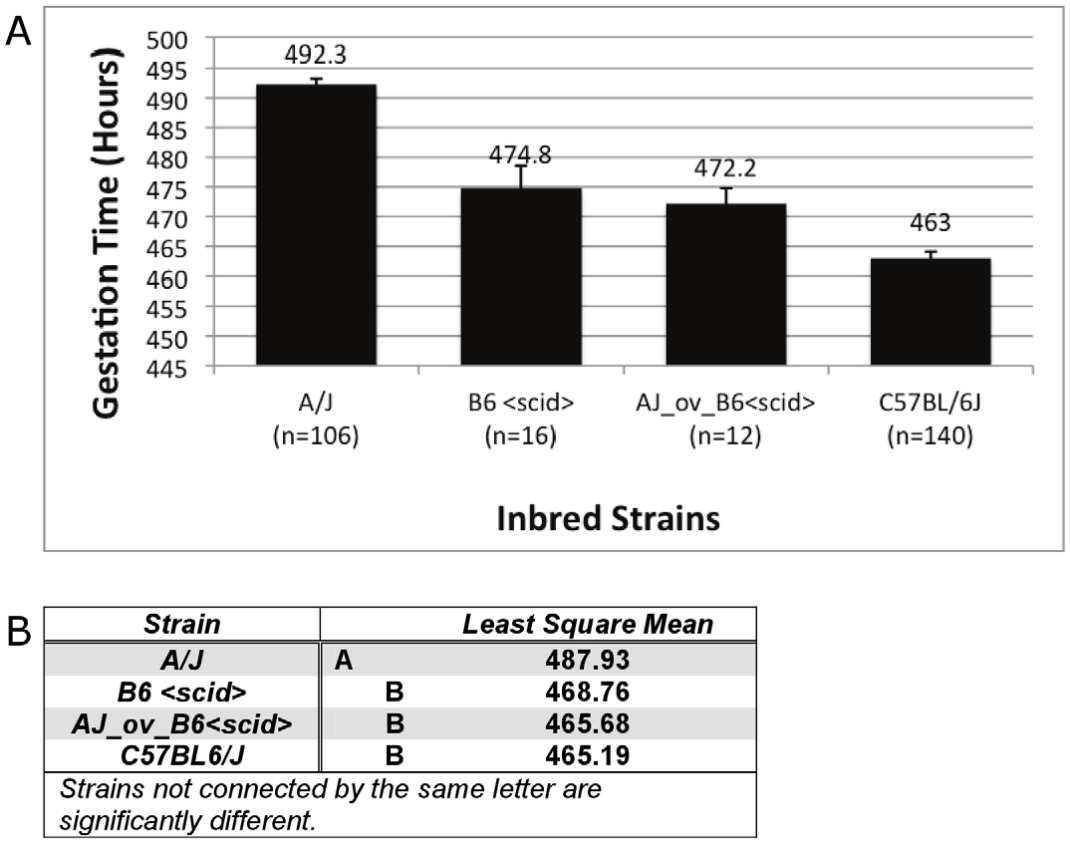
Gestation length is primarily dependent upon maternal genotype. (A) Gestation length presented in hours of C57BL/6J, A/J, B6.CB17-*Prkdc^scid^*/SzJ (B6-*scid*), and B6-scid with transplanted A/J ovaries (AJ-ov-B6-*scid*). In order to directly compare the effect of pure B6 and A/J pups in a B6 maternal background, B6-*scid* mice were sham manipulated and mated to B6-scid males, while AJ-ov-B6-*scid* females were mated to A/J males following recovery from ovary transplant (see materials and methods for details). (B) Graphical representation of strains with significantly different GLs independent of all other factors. Strains that are not associated by a letter are significantly different from each other (p<0.05), demonstrating that B6 females show no statistical difference in gestation time, regardless of the genotype of the pups.

Although our data strongly supports a genetic role in determining GL, it is not clear the degree to which this effect depends on the maternal genotype, and whether the genetic background of the pups has any impact. To address this question, we performed ovary transplant experiments in which A/J ovaries were transplanted into ovaryectomized B6.CB17-*Prkdc^scid^*/SzJ (B6-*scid*) immunocompromised mice [8], and the GL compared to both B6 and B6-*scid* mice. In each case, the females were mated to males of the genetic background matching the transferred ovary, which allowed us to directly assess the effect of pure A/J pups in the context of a B6 maternal background. Our results clearly show that in all cases, B6 females, regardless of the genotype of their pups, show statistically similar GLs, dramatically different from A/J (Figure 2). The slight, though not statistically significant, difference between B6 and B6-*scid* or AJ-ov-B6-*scid* is likely due to the fact that B6-*scid* mice tend to have smaller litters than B6 mice, resulting in a marginal lengthening of GL. Although we could not perform the converse experiment since A/J-*scid* or a similarly immunocompromised strain is not available, our data strongly supports the primary role of maternal genetic makeup in the determination of GL.

**Figure 3:**
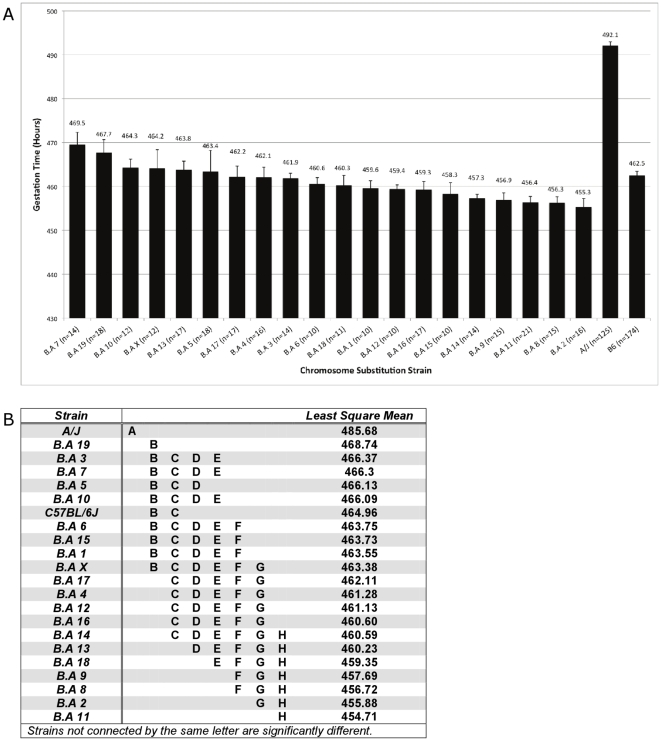
Complex, polygenic regulation of gestation length is revealed by the B.A CSS panel. (A) Mean gestation time in total hours for each of 20 CSS. The total number of pregnancies monitored is indicated for each strain and data are presented as the mean +/− S.E.M. (B) Graphical representation of individual CSS with significantly different GLs independent of all other factors. Strains that are not associated by a letter are significantly different from each other (p<0.05).

To begin the process of elucidating the genetic loci that underlie the A/J-B6 GL difference, we used the B.A CSS panel, a powerful set of tool strains wherein individual A/J chromosomes have been bred into a pure B6 background [5]. By measuring the GL of each individual strain, we sought to narrow the causative loci to a chromosome or smaller set of chromosomes. As shown in Figure 3, the results reveal a much more complex relationship between genetic background and GL than previously anticipated. In general, the GL of the CSS set is clustered around that of B6, suggesting no individual strain captures a large proportion of the A/J effect. Surprisingly, six strains show significantly shorter GL (B.A-Chr13, B.A-Chr18, B.A-Chr8, B.A-Chr2, B.AChr9 and B.A-Chr11) versus B6 control. These data suggest a complex interplay between multiple loci in the regulation of GL, which will require further approaches to identify individual genes that regulate parturition. However, these data will allow us to prioritize the use of recombinant inbred (RI) lines to further narrow intervals harboring loci that control GL.

## Discussion

Our data clearly indicate that genetic background plays a major role in determining gestation length, and supports the use of mice to study the genetic regulation of preterm birth. Given the large difference between C57BL/6J and 129S1/SvImJ, this information is particularly relevant to investigators utilizing knockout models, which are frequently maintained on segregating backgrounds, to understand mechanisms that govern preterm birth [3], [4], [9]. Similarly, this information will be critical for the study of perinatal development and organogenesis by providing a more definitive total GL.

There is significant debate in the literature as to the contribution of paternal (and by extension, fetal) genetics to preterm birth risk [10]. While it is clear that fetal-maternal communication is essential to parturition, most genetic studies confirm the primary role of the mother in preterm birth risk and gestational timing [11], [12]. Our study demonstrates that the same principle holds true for mouse GL, as B6 females with A/J pups exhibit a GL similar to that of B6 females with B6 pups. This does not discount the strong correlation with litter size, which clearly affects GL, but indicates that maternal genetic factors alone also account for differences in GL in mice. In addition, our study excludes the ovary, which is the source of progesterone, as the cause of maternal genetic variation. This is important as human parturition does not involve a simultaneous drop in progesterone levels as is seen in the mouse, suggesting the genes underlying the strain differences observed in this study may be directly relevant to preterm birth in the human population.

By identifying a firm genetic basis for GL independent of litter size or pregnancy load, we establish a foundation for the identification of genes and pathways that regulate GL and timing of parturition, which clearly involves multiple interacting loci. Indeed the results from our B.A CSS experiments are an excellent demonstration of that complexity, which will require more sophisticated genetic approaches to uncover. We have already begun to measure the GL of AXB and BXA recombinant inbred strains [5], which should provide increased power and mapping resolution over the CSS approach alone. We are prioritizing the lines based on our CSS data, focusing on those RI lines with multiple recombinations on Chromosomes 2, 8, 9, 11, 13 and 18). We have also begun the process of creating nested congenic lines to further narrow the interval for the hit on chromosome 11, our most robust finding. Recently, a SNP in the gene *timp2*, the mouse orthologue of which resides on mouse chromosome 11, has been associated with preterm birth in a human population [13]. Our ovary transplant experiments further suggest that both extended crosses from RI lines (RIX) and traditional crosses are feasible, given the relatively small fetal contribution to GL, beyond litter size. These data therefore not only support the use of large-scale genome wide association studies in humans to identify determinants of preterm birth, but also promise to complement these studies through the combined power of comparative genetics.

## Materials and Methods

### Mice

All animal experiments have been conducted according to relevant national and international guidelines (AALAC and IACUC) and have been approved by the Jackson Laboratory Animal Care and Use Committee (Genetic Resource Science protocol #99066). For each inbred strain 20 females and 10 males between 6–8 weeks of age were obtained from production or repository colonies at the The Jackson Laboratory (Bar Harbor, ME). All mice were housed in pressurized, individually ventilated (PIV) racks (Thoren Caging Systems Inc, Hazleton, PA) on bedding of white pine shavings. Each pen contained a wire-rod metal top that holds feed pellets and a water bottle. Mice had access to bottled acidified water (pH 2.8–3.1) and NIH 31M feed pellets with 6% fat (Purina Mills Inc., Richmond, IN) *ad libitum*. The light∶dark cycle was maintained at 12∶12. In order to eliminate variability that might arise from first litters, all females were mated and allowed to deliver one litter before being placed into the queue for gestation time monitoring. Following first litters, males and females from each strain were moved simultaneously into a separate room outfitted with monitoring equipment and infrared lighting and allowed to acclimate for at least one week prior to being mated.

### Matings and pregnancy monitoring

Twice a week, pair matings were set at the end of the day, and females checked early the following morning for the presence of a vaginal plug to indicate a successful mating. Fertilization (0 hour) was calculated to be the midpoint of the dark cycle prior to the appearance of the copulation plug. Pregnant females were weighed and housed separately from the male until monitoring. At E14.5 females were again weighed and housed singly in pens containing bed-o-cob bedding, which cannot be piled so as to obscure the view of the cameras. Females were monitored continuously from E14.5 on, using infrared lighting and video cameras (Inter-Pacific, Wheeling, IL) until the appearance of the first pup. Following delivery, mother and pups were moved to a pen containing white pine shavings, and the litter was weighed as a group. Each video was examined for the precise time of birth, determined by the appearance of the first pup. Data collected included: total gestation time, size (weight and number) of each litter, female weight gain as an indication of the total “pregnancy load,” and pup mortality. Variability in the total number of females measured is due to differences in reproductive performance or morbidity associated with characteristics of the individual strain, such as lymphoma in the AKR/J strain and diabetes in NOD/ShiLtJ.

### Ovarian transplants

Transfer of A/J ovaries to B6.CB17-*Prkdc^scid^*/SzJ mice was performed as described [8] and in accordance with IACUC guidelines. Briefly, donor A/J female mice are euthanized by cervical dislocation and ovaries dissected free of the fat pad and bursa in cold, sterile saline. Recipient B6.CB17-*Prkdc^scid^*/SzJ mice are anesthetized and both ovaries are carefully removed from the bursa and fat pad. The donor A/J ovary is then placed into one of the recipient B6.CB17-*Prkdc^scid^*/SzJ bursa in contact with the severed ovarian blood vessels. The recipient mice are then sutured and allowed to recover before being mated to A/J males. Successful removal of recipient ovaries is monitored by coat color of pups.

### Statistics

Anova analysis, effects tests, and pair-wise correlations were conducted with the JMP software (SAS Institute, Cary, NC). Significance was set at p<0.05.
